# Intramural ectopic pancreatic tissue of the stomach: A case report of an uncommon origin of a non-cancerous gastric tumour

**DOI:** 10.1016/j.ijscr.2020.06.081

**Published:** 2020-06-23

**Authors:** Enrica Chiriatti, Paulina Kuczma, Domenico Galasso, E. Koliakos, Edgardo Pezzetta, Olivier Martinet

**Affiliations:** a Department of General Surgery, Hospital Riviera – Chablais (HRC), Site of Montreux, Avenue de Belmont 25, 1820 Montreux, Switzerland; b Department of General Surgery, Hospital of Valais (CHVR), Avenue du Grand-Champsec 80, 1951 Sion, Switzerland

**Keywords:** Case report, Heterotopic pancreas, Abdominal surgery, Gastric tumour, Pancreas, Gastrointestinal surgery

## Abstract

**Introduction:**

Ectopic pancreatic tissue is often incidentally encountered during abdominal surgery. We report a case of an incidental finding during a laparoscopic appendectomy, the approach to diagnosis and management of this.

The work has been reported in line with the SCARE criteria.

**Presentation of case:**

A 32 year-old woman was diagnosed with an intramural pre-pyloric mass during a laparoscopic appendectomy. The lesion was identified on a subsequent computed tomography as a homogenous mass on the greater curvature. Upper esophagho-gastro-duodenoscopy was normal. The intramural mass was confirmed by an endoscopic sonography and the fine needle biopsy showed aspecific inflammatory cells. A laparoscopic wedge resection was realized. Histopathologic examination confirmed the diagnosis of an ectopic pancreatic tissue.

**Discussion:**

The management of ectopic pancreas poses a medical challenge. The diagnostic quiver consists of radiologic exams and endoscopy, in combination with a direct biopsy of the lesion.

**Conclusion:**

Despite the plethora of diagnostic modalities available, a definitive diagnosis for heterotopic pancreas often remains elusive, requiring more invasive diagnostic means. Although ample information is available in literature, there are currently no evidence-based guidelines regarding diagnosis and management of heterotopic pancreas.

## Highlights

• Ectopic pancreatic tissue is a rare incidental finding during abdominal surgery.
• 80% of the lesions are unifocal and less than 3 cm in size.
• The most frequent site is the stomach, followed by duodenum and proximal jejunum.
• The imaging modalities and endoscopic biopsy often remain inconclusive.
• Only resection and and histopathologic examination provide the definitve diagnosis.

## 1 Introduction

Ectopic pancreas is defined as the presence of pancreatic material in tissues where it is normally absent, that has no anatomic, vascular or ductal continuity with the orthotopic gland [1]. Although the exact pathogenesis remains unknown, multiple theories attempt to explain its embryological origin, the most prevalent of which being the misplacement theory. According to the misplacement theory, fragments of pancreatic tissue deposit onto adjacent loci during organogenesis and later develop into mature elements [2]. This theory may explain the majority of cases in which the ectopic pancreatic tissue is located in other organs derived from the primitive foregut, but it does not explain other occurrences like in Meckel’s diverticulum or the colon [3,4]. Alternative theories include the metaplasia theory, according to which endodermal tissues migrate to the submucosa during embryogenesis and develop into pancreatic tissue [1,4] and the totipotent theory in which totipotent endodermal cells lining the gut differentiate into pancreatic tissue [1,4,5].

Structurally the ectopic pancreatic tissue resembles the orthotopic pancreas in gross and microscopic anatomy and appears as a firm, intramural nodular mass [1,6]. A classification system initially developed by Heinrich in 1909 and later modified by Fuentes in 1973 [7], divides the ectopic pancreatic tissue into four histologic types (Table 1).

**Table 1:** Original and modified histologic classification of heterotopic pancreatic tissue.

Type	Histologic features (Heinrich)	Histologic features (modified by Fuentes)
Type 1	Normal pancreatic tissue components present, including acini, ducts and islets of Langerhans	Normal pancreatic tissue components present, including acini, ducts and islets of Langerhans
Type 2	Incomplete or lobular arrangement (few acini and multiple ducts). Absence of endocrine elements.	Presence of ducts only.
Type 3	Presence of ducts; acini and islet cells are absent.	Exocrine pancreas - acini only.
Type 4	–	Endocrine pancreas - islet cells only.

Most lesions are unifocal (80%) and up to 80% are reported to be less than 3 cm in size [8]. Its prevalence in autopsies ranges from 0.55% to 13.7% [2]. Literature suggests that the most frequent site is the stomach with a prevalence ranging from 38% to 86%, with duodenum (36%) and proximal jejunum (16%) also being common sites of presentation. Other less common locations include Meckel’s diverticulum and ileum [6,9,10] while extra-digestive locations have also been described. Although ectopic pancreatic tissues are usually asymptomatic and are discovered as incidental findings, they may present symptoms, the most common of which are nausea and vomiting (27%), epigastric pain (27%) and weight loss (18%) [11] as well as a variety of less common symptoms depending on their location, such as gastric obstruction by a pyloric mass, intussusception of a jejunal lesion or biliary obstruction by a lesion at the ampulla of Vater. The most common complaint is abdominal pain.

In this case report, we present the case of a woman with a gastric ectopic pancreatic tissue located incidentally during a laparoscopic appendectomy in our community hospital, showcasing the management process of this unexpected perioperative finding.

## 2 Presentation of case

A 32 year-old woman with no significant medical history was admitted to our hospital presenting symptoms of lower abdominal quadrant pain. Physical examination revealed a soft abdomen with tenderness located at the right iliac fossa. Blood tests showed a mild leucocytosis and a moderate elevation of C-reactive protein. The diagnosis of appendicitis was confirmed with an ultrasound of the lower abdomen.

A decision was made to proceed with an immediate laparoscopic appendectomy. During the exploration of the abdominal cavity, a pre-pyloric 3 cm mass was located along the greater curvature of the stomach. An appendectomy was performed and the decision to investigate the mass postoperatively was made.

**Fig. 1:**
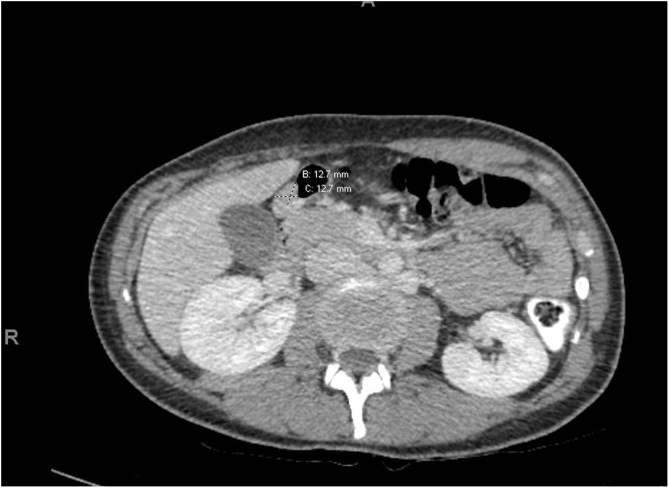
Abdominal CT scan showing the pre-pyloric, homogenously enhancing mass. The lesion has similar appearance as the nearby normal pancreatic tissue.

**Fig. 2:**
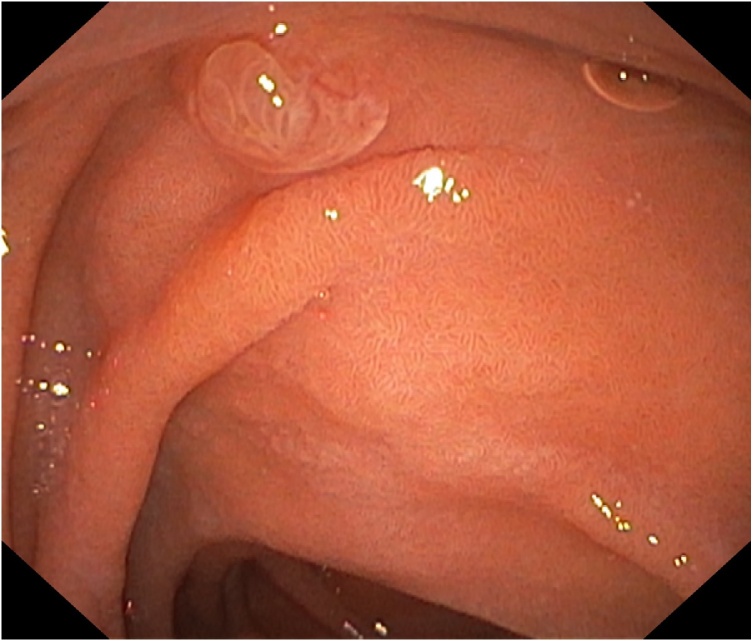
Esophagogastroduodenoscopy image demonstrating the partially exophytic lesion near the great gastric curvature.

**Fig. 3:**
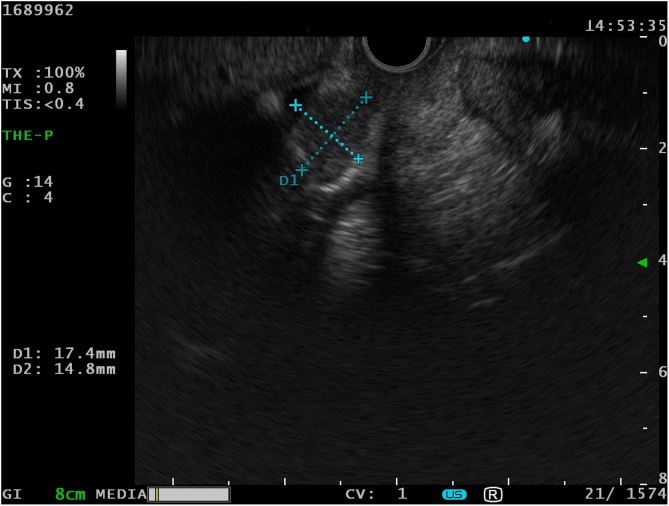
Endoscopic ultrasound image showing the hypoechoic mass of 17.4 mm × 14.8 mm.

A subsequent computed tomography (CT) scan showed a homogenously enhancing mass of 12.7 × 12.7 mm (Fig. 1), while an upper esophago-gastro-duodenoscopy (EGD) showed a partially exophytic lesion on the greater gastric curvature (Fig. 2). The presence of the mass was confirmed in an upper GI endoscopic ultrasound (Fig. 3). Fine needle biopsy was inconclusive, showing aspecific inflammatory cells. In this setting, a decision to return to the operating theatre was made, in order to perform a laparoscopic wedge resection for diagnostic and therapeutic purposes. The postoperative period was uneventful and the patient was discharged on day 3 [17].

**Fig. 4:**
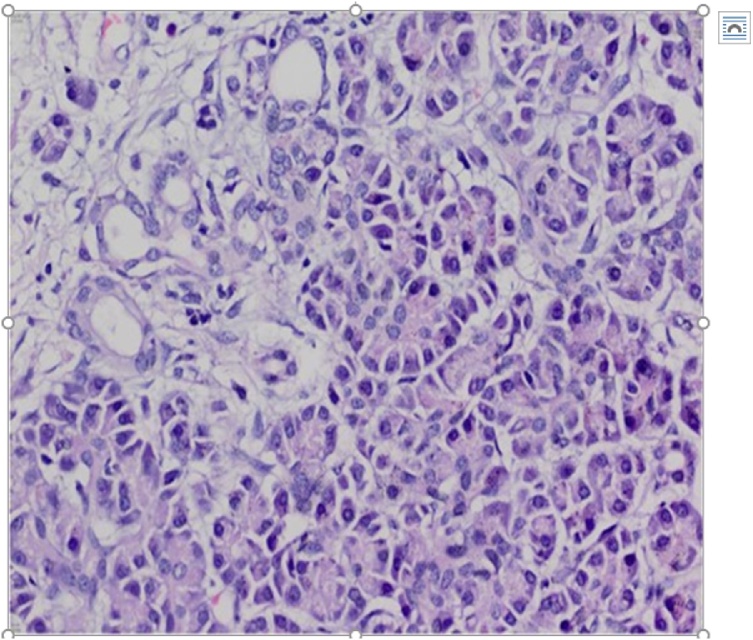
Histopathology showing pancreatic acinar cells and pancreatic ducts in the ectopic pancreatic tissue.

Histopathologic examination revealed a heterotopic pancreatic tissue within the gastric wall (Fig. 4) with normal pancreatic tissue components present, including acini, ducts and islets of Langerhans.

## 3 Discussion

To this day the management of ectopic pancreas poses a medical challenge. Although an abundance of information on the nature of the disease exists in the literature, as well as pertinent statistical data (prevalence, lesion location, malignancy potential, diagnostic efficacy of radiologic tools, etc.) the source of this knowledge is mainly case reports or retrospective studies of small patient pools, resulting into great statistical variance and inconsistency.

Despite the evolution of diagnostic modalities, there is no golden standard for the diagnosis of this lesion nor a defined protocol to facilitate it exists. The diagnostic quiver consists of radiologic exams and endoscopy, in combination with a direct biopsy of the lesion.

Radiologic tools that may be employed include upper GI series (UGI), ultrasound (US) and endoscopic ultrasound (EUS), computed tomography (CT), magnetic resonance imaging (MRI) and MR cholangiopancreatography (MRCP). Radiologic findings in UGI, US and CT are well defined, while documentation of ectopic pancreatic tissue characteristics in MRI remain limited [12]. Despite of the many modalities available, radiologic tools may be suggestive of the diagnosis, but remain unable to preclude an invasive diagnostic procedure.

Gottschalk U et al. mention in their study that the use of EUS, although helpful in the diagnosis of subepithelial lesions, has limited efficacy in differentiating an ectopic pancreas from other lesions with similar appearance such as gastrointestinal stroll tumours (GISTs), submucosal neuroendocrine tumours, hamartomas and schwannomas. They also note a discordance in literature regarding the echographic characteristics of the ectopic pancreas [13]. A potential advantage of EUS is the ability to perform a fine-needle biopsy.

Endoscopy of the upper GI tract is a tool with high sensitivity but low specificity for the diagnosis of ectopic pancreas. In EGD, the ectopic pancreatic rest appears as a submucosal mass with endoluminal growth pattern, while central umbilication can be noticed owning to the presence of a rudimentary ductal system. Due to the subepithelial nature of the lesion, superficial endoscopic biopsy is inadequate [1].

Current data are unable to indicate a standardised and cost effective diagnostic procedure. Despite the modern imaging capabilities, the basis of the diagnosis of a gastric heterotopia remains the wedge resection of the lesion and histopathologic examination of the specimen [16]. Furthermore, there is currently no consensus on a potentially conservative management of a heterotopic pancreatic tissue. On the other hand, ectopic pancreatic tissue can be life-saving for patients who need a total pancreatectomy for cancer or chronic pancreatitis. In this context, it would seem reasonable to leave the ectopic pancreatic tissue in place to prevent a surgery-induced diabetes. An accumulation of non-invasive diagnostic methods could help in the future to identify a pancreatic ectopia and to preserve it in this cohort of patients.

In this case report we present a patient with a pre-pyloric mass incidentally found during a laparoscopic appendectomy. Despite of a series of exams that were performed, the lack of diagnosis posed the indication to return the patient to the operating theatre for a laparoscopic wedge resection of the lesion [14].

## 4 Conclusion

Heterotopic pancreas is a rare medical entity that is most commonly encountered as an incidental finding during a radiological test or perioperatively [15]. Clinical as well as radiological presentation are unspecific and often prove inconclusive, requiring more invasive diagnostic tools to be employed. A pancreatic heterotopia should be included in the differential diagnosis of upper GI tract masses. High clinical suspicion is necessary. At this time, no evidence-based guidelines for the management of a heterotopic pancreatic tissue exist. While the majority of cases are benign, heterotopic pancreatic loci may harbour malignant potential. This case report highlights this rare perioperative incidental finding and the necessity of systematic studies in order to form a consensus on the diagnostic process and management of these lesions.

## Declaration of Competing Interest

There is no conflict of interests. None of the authors received no funding whatsoever except the regular salary from the hospital where they are employed.

## Funding

There was no funding nor any sponsors involved.

## Ethical approval

The case report is exempt from ethical approval. No study was conducted; we report state-of-the art diagnostic work-up and treatment of a rare case for which the approval of the ethics committee was not needed.

## Consent

Written informed consent was not obtained from the patient but she gave us her consent by call. Because we were unable to receive the written consent from the patient, the head of our medical team has taken responsibility and confirms that an oral consent by phone call was obtained and that the paper has been sufficiently anonymised not to cause harm to the patient or their family. A copy of a signed document stating this is available for review by the Editor-in-Chief of this journal on request.

## Author contribution

All of the authors contributed to the data collection, analysis and interpretation and paper writing.

Enrica Chiriatti (the First author) was responsible to : writing paper, study concept, data collection, data analysis.

Paulina Kuczma: contributed to study design, communication with the Editor.

Domenico Galasso: contributed to data collection.

Evangelo Koliakos: contributed to study design.

Edgardo Pezzetta: contributed to data analasys.

Olivier Martinet: contributed to study concept.

## Registration of research studies

The following information can be found on the website http://www.researchregistry.com: We do not register case reports that are not first-in-man or animal studies.

This is our case: it is a case report that is not a first-in-man study.

## Guarantor

Enrica Chiriatti, MD.

## Provenance and peer review

Not commissioned, externally peer-reviewed.
